# Pharmacokinetics, efficacy and tolerance of cefoxitin in the treatment of cefoxitin-susceptible extended-spectrum beta-lactamase producing *Enterobacterales* infections in critically ill patients: a retrospective single-center study

**DOI:** 10.1186/s13613-022-01059-9

**Published:** 2022-09-30

**Authors:** Paul Chabert, Judith Provoost, Sabine Cohen, Céline Dupieux-Chabert, Laurent Bitker, Tristan Ferry, Sylvain Goutelle, Jean-Christophe Richard

**Affiliations:** 1 Hospices Civils de Lyon, Médecine Intensive – Réanimation, Hôpital de La Croix Rousse, 103 Grande rue de la Croix Rousse, 69004 Lyon, France; 2 Hospices Civils de Lyon, Maladies Infectieuses et Tropicales, Hôpital de La Croix Rousse, 103 Grande rue de la Croix Rousse, 69004 Lyon, France; 3 Unité Fonctionnelle de Pharmacologie Spécialisée, Hospices Civils de Lyon, UM de Pharmaco-Toxicologie, Centre Hospitalier Lyon Sud, 165 Chemin du Grand Revoyet, 69495 Pierre-Bénite Cedex, France; 4 Hospices Civils de Lyon, Institut Des Agents Infectieux, Hôpital de La Croix Rousse, 103 Grande rue de la Croix Rousse, 69004 Lyon, France; 5 Université de Lyon, 92 rue Pasteur, CS 30122, 69361 Lyon Cedex 07, France; 6 Université Claude Bernard Lyon 1, 43 Boulevard du 11 Novembre 1918, 69100 Villeurbanne, France; 7 Service de Pharmacie, Groupement Hospitalier Nord, Hospices Civils de Lyon, Lyon, France; 8 UMR CNRS 5558, Laboratoire de Biométrie et Biologie Evolutive, Université de Lyon, Université Claude Bernard Lyon 1, Villeurbanne, France; 9 CREATIS UMR 5220, INSA-Lyon, CNRS, INSERM, U1294, Université de Lyon, Université Claude Bernard Lyon 1, 69621 Lyon, France

**Keywords:** Antibacterial chemotherapy, Intensive care, Healthcare-associated pneumonia, Extended-spectrum beta-lactamase, Carbapenem-sparing agents, Cefoxitin, Population pharmacokinetics

## Abstract

**Background:**

Cefoxitin is active against some extended-spectrum beta-lactamase-producing *Enterobacterales* (ESBL-PE), but has not been evaluated so far in the intensive care unit (ICU) settings. Data upon its pharmacokinetics (PK), tolerance and efficacy in critical conditions are scanty. We performed a retrospective single-center study in a university hospital medical ICU, in subjects presenting with cefoxitin-susceptible ESBL-PE infection and treated with cefoxitin. The primary aim was to determine cefoxitin PK. Secondary endpoints were efficacy, tolerance, and emergence of cephamycin-resistance.

**Results:**

Forty-one patients were included in this study, mainly with ESBL-PE pneumonia (35 patients, 85%). Cefoxitin was administered during a median [interquartile range (IQR)] duration of 5 [4–7] days. Cefoxitin serum concentrations strongly depended on renal function. Target serum concentration (> 5 × minimum inhibitory concentration (MIC) 24 h after cefoxitin onset was obtained in 34 patients (83%), using a median [IQR] daily dose of 6 [6–6] g with continuous administration. The standard dosage of 6 g/24 h was not sufficient to achieve the PK/PD target serum concentration for MIC up to 4–8 mg/L, except in patients with severe renal impairment and those treated with renal replacement therapy. Treatment failure occurred in 26 cases (63%), among whom 12 patients (29%) died, 13 patients (32%) were switched to alternative antibiotic therapy and 11 patients (27%) presented with relapse of infection with the same ESBL-PE. Serious adverse events attributed to cefoxitin occurred in 7 patients (17%). Acquisition of cephamycin-resistance with the same *Enterobacterales* was identified in 13 patients (32%), and was associated with underdosage.

**Conclusion:**

Continuous administration of large doses of cefoxitin appears necessary to achieve the PK/PD target in patients with normal renal function. Renal status, MIC determination and therapeutic drug monitoring may be useful for treatment individualization in this setting. The treatment failure rate was 63%. The cefoxitin safety profile was favorable, but we observed a high rate of cephamycin-resistance emergence.

**Supplementary Information:**

The online version contains supplementary material available at 10.1186/s13613-022-01059-9.

## Background

Multiresistant bacteria are a major public health issue [1]. The rate of extended-spectrum beta-lactamase-producing *Enterobacterales* (ESBL-PE) carriage has recently reached 18% in French hospitals [2]. They are responsible for an increasing rate of nosocomial infections in the intensive care units (ICU) [3, 4]. Their treatment often requires broad-spectrum antibiotics, such as carbapenems, whose use increases the risk of selecting resistant strains such as carbapenemase producing *Enterobacterales* [5, 6]. Antibiotic stewardship in the ICU supports the use of broad-spectrum antibiotic-sparing strategies [7], and restricting the use of carbapenems has been recommended [8, 9].

A possible alternative to carbapenems for the treatment of susceptible strains of ESBL-PE infections is cefoxitin, a cephamycin that circumvents the enzymatic degradation by ESBL. Cefoxitin has long been known as effective against some multiresistant bacteria [10], making it an appealing carbapenem-sparing candidate drug for ESBL-PE infections [11, 12]. Its clinical use is deemed relevant in many infectious situations [13], such as urinary tract infections in non-ICU patients [14–17]. Studies assessing the efficacy of cefoxitin for other indications are scarce [18]. Some studies reported the usefulness and tolerance of other cephamycins such as cefmetazole in the treatment of ESBL-PE bacteremia [19, 20]. Very few studies have described the toxicity of cefoxitin in humans, mostly in the perioperative prophylactic setting [21]. Cefoxitin’s ecological impact might not be negligible as acquisition of cefoxitin-resistance has been reported [22–25]. Finally, the use of cefoxitin in the ICU settings has not been evaluated so far.

The primary aim of the present study was hence to determine the pharmacokinetics/pharmacodynamics (PK/PD) and dosage requirements of cefoxitin in ICU patients. Secondary aims were to assess the efficacy of cefoxitin in ESBL-PE infections, its tolerance and its impact on acquisition of cephamycin-resistance inside the ICU.

## Methods

This was a single-center retrospective cohort study performed between 1 January 2014 and 1 June 2021 in a university teaching hospital medical ICU. Follow-up was completed on 31 December 2021.

### Inclusion and exclusion criteria

Subjects were included if they met all the following criteria: age ≥ 18 years, infection with cefoxitin-susceptible ESBL-PE, treatment with cefoxitin during ≥ 24 h as a definitive antibiotherapy regardless on the probabilistic antibiotherapy previously administered, and cefoxitin therapeutic drug monitoring (TDM) with at least one serum concentration measured during therapy. Cefoxitin was administered preferentially with continuous infusion unless clinician decided otherwise. Infection was defined by the presence of clinical abnormalities that were related by clinicians to the microbiological findings and for which an antibiotherapy was initiated. Inclusion started at the initiation of cefoxitin treatment. Patients who refused to participate or were included in the study during a previous ICU stay were excluded.

### Data collection

Data were retrospectively collected from the medical charts. The follow-up was right-censored 30 days after inclusion. Comorbidity was assessed using Charlson index [26]. Patients were considered immunocompromised if they received ≥ 20 mg prednisone/24 h for > 1 month, had received a solid-organ or hematopoietic stem cell transplant, were diagnosed with malignant hemopathy, were taking an immunosuppressive drug (anti-calcineurin, mycophenolate mofetil, methotrexate, azathioprine, tumor necrosis factor α-blocker, interleukin-6-blockers or B-cell depletion), received cancer chemotherapy within 6 months, or had human immunodeficiency virus infection with ≤ 200 CD4 cells/µL. The following risk factors for multiresistant bacteria carriage were considered: current hospital stay duration > 5 days [27], previous antibiotic exposure within 3 months before diagnosis of ESBL-PE infection, previous ESBL-PE carriage or infection within 3 months, travel abroad in high community ESBL-PE prevalence area within 3 months before inclusion, living in a long-term care facility [9]. Patient could be treated with a probabilistic antibiotic prior to inclusion. The empiric treatment was considered adapted if at least one antibiotic was retrospectively active against the strain identified. The effective modalities of cefoxitin treatment were registered (continuous or discontinuous infusion, dosages, duration of treatment). Illness severity at the time of ICU admission and at inclusion was assessed using Sequential Organ Failure Assessment (SOFA) [28] items, Simplified Acute Physiology Score (SAPS) II [29], and sepsis criteria [30]. Glomerular filtration rate was estimated using the Cockcroft–Gault equation [31].

### Biological analysis

The total serum cefoxitin concentration was measured using high-performance liquid chromatography with high-resolution mass spectrometry detection using a QExactive Focus Orbitrap (Thermo Fisher Scientific, Waltham, USA). The presence of the molecule was based on the mass of its parent ion with an accuracy of 5 ppm and on its retention time. The quantification was based on the principle of internal calibration using cefazolin-[13]C2[15]N as internal standard. This method was validated over a range of 0.5 to 100 mg/L. Each batch of patient analysis includes low- and high-quality controls, respectively, at 10 mg/L and 50 mg/L. The intra- and inter-assay variability of the quality controls was lower than 15% at both levels. This analytical method is available in routine analysis in our institution and is validated according to accreditation recommendations in medical biology (French accreditation committee, COFRAC).

All biological samples positive for cefoxitin-susceptible ESBL-PE sent for bacteriological analysis were considered if the amount of bacteria exceeded the threshold defined for the sample site [32, 33]. ESBL-PE were suspected based on the antibiotic susceptibility testing, and cefoxitin-susceptibility was assessed following the latest European and French guidelines [34]. Minimum inhibitory concentration (MIC) was estimated with Vitek 2 (BioMérieux, Marcy l'Étoile, France). MIC was systematically re-assessed with *E-*test when MIC Vitek2 estimation was between 4 mg/L and the epidemiological cut-off (MIC_ECOFF_) value 8 mg/L, or upon request from clinicians.

### Primary and secondary endpoints

The primary endpoint was the achievement of cefoxitin PK/PD target serum concentration of efficacy based on PK modeling. This target was defined as 100% of time spent above the MIC for the modeled free serum concentration (*f*T_>MIC_) on the second day of therapy. This was considered equivalent to a steady-state total cefoxitin serum concentration (C_ss_) > 5 × MIC in the case of a continuous administration, assuming a cefoxitin protein binding of 80% [35–37]. Secondary endpoints were the following: (1) the rate of treatment failure defined as a composite endpoint: change of antibiotic treatment before full treatment course, or relapse of infection with the same micro-organism, or death within 30 days after inclusion; (2) change of antibiotic treatment before full cefoxitin treatment planned course; (3) relapse of infection with the same micro-organism (defined as new onset of symptoms compatible with infection, microbiological growth with the same micro-organism, and introduction of a new antibiotic treatment) before day-30; (4) mortality at day-30 after inclusion; (5) attributed cause of the criteria of failure definitely linked to the initial ESBL-PE (death, relapse, or change of antibiotic associated with microbiological analysis performed after inclusion showing one or more positive culture to the same initial ESBL-PE) or possibly linked (death, or change of antibiotic and microbiological analysis showing no positive culture, and no alternative infectious cause identified) or not linked (death, or change of antibiotic associated with microbiological analysis positive with another strain explaining the treatment failure or non-infectious cause of failure) (in the case of multiple criteria of failure in a same patient, failure was definitely linked to the initial ESBL-PE if at least one criteria of failure was definitely linked, it was possibly linked to the initial ESBL-PE if at least one criteria of failure was possibly linked and no criteria was definitely linked, and it was not linked to the initial ESBL-PE if no criteria was definitely nor possibly linked); (6) occurrence of serious adverse event under cefoxitin treatment including but not limited to: rash, hepatic cytolysis or cholestasis (> 3 N or 3 times increase compared to values at inclusion), acute kidney injury (any increase of ≥ 1 point in KDIGO [38] staging under cefoxitin treatment), encephalopathy attributed to cefoxitin by clinicians, treatment discontinuation or change of antibiotic due to cefoxitin suspected intolerance; (7) rate of acquisition of cephamycin-resistance with the same microbial species in any biological sample performed between the initiation of cefoxitin (bacterial carriage or infection) and day-30 after inclusion in samples for which an antibiotic susceptibility testing was performed; (8) *Clostridioides difficile* infection before day-30 after inclusion.

### Statistical analysis

Data were expressed as median [quartile 1–quartile 3] for quantitative variables, and count (%) for qualitative variables. Normality distribution was assessed with the Schapiro–Wilk test and with quantile–quantile plots. Qualitative variables were compared with exact Fisher test. Quantitative variables were compared using Student’s *t*-test or Wilcoxon–Mann–Whitney test. The statistical significance threshold was set to a *p*-value < 0.05 using bilateral tests. Analyses were performed using STATA^®^ (version 12, StataCorp, College Station, Texas, USA) and R software (version 4.1.1, R Foundation for Statistical Computing, Vienna, Austria) with package fitdistrplus [39]. Since some MIC were only evaluated with Vitek2 and hence were left censored below a low threshold value (i.e., 4 mg/L), they were imputed as follows: (1) cefoxitin MIC of 30 ESBL-PE (*Escherichia coli, Klebsiella pneumoniae, Klebsiella oxytoca,* and *Citrobacter koseri*) assessed with E-test in our microbiological laboratory were fitted by maximum likelihood to a log-normal distribution [39]; (2) 30,000 values were simulated under the log-normal distribution with fitted parameters; (3) censored values were then replaced with a number randomly selected from a subset of the simulated dataset restricted to values below 4 mg/L.

Population PK analysis was carried out by using the Monolix^®^ software (version 2019R2, Lixoft, Antony, France). The best structural and covariate model was selected based on classical criteria (goodness-of-fit, precision of parameter estimates, and simulation-based diagnostics). In covariate modeling, the following variables were examined: age, sex, total body weight (TBW), ideal body weight (IBW), adjusted body weight (ABW), creatinine clearance (CCR) estimated by the Cockcroft–Gault equation based on either TBW, IBW or ABW, renal replacement therapy (RRT, coded as a binary variable), SAPS-II, SOFA, and serum protein. The achievement of the target cefoxitin serum concentration was determined based on model predictions (PK profile based on the Bayesian posterior estimates). Then, the final model was used to perform 1000-subject Monte Carlo simulations with the Pmetrics^®^ program [40] to identify optimal dosing regimens for continuous IV cefoxitin. We simulated continuous IV administration of cefoxitin with daily doses ranging from 2 to 12 g, after a loading dose of 2 g administered over 1 h. As renal function was found to influence cefoxitin clearance, each dosing regimen was simulated in patients with various level of renal function (CCR based on IBW, CCR_IBW_) ranging from 10 to 200 mL/min. We considered a MIC range of 0.125 to 16 mg/L and an MIC_ECOFF_ of 8 mg/L, in accordance with the cefoxitin MIC distributions of *Enterobacterales* from EUCAST. The probability of target attainment (PTA) was computed, with PTA ≥ 90% considered as acceptable.

## Results

### Description of the cohort

**Fig. 1:**
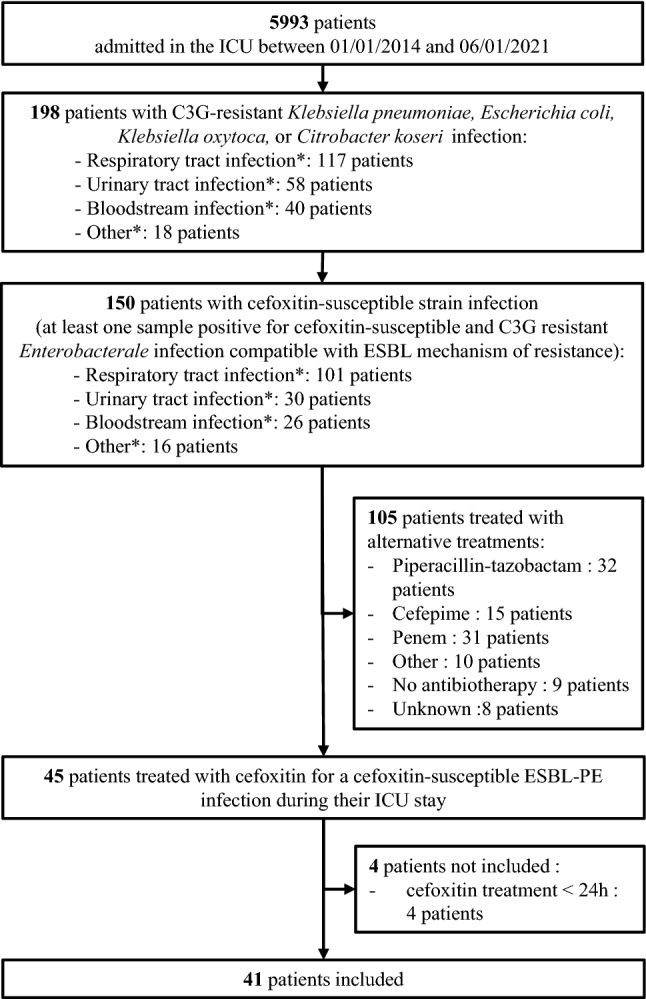
Flowchart of the study. ^*^Multiple sites could be positive for ESBL-PE per patients. *C3G* 3rd generation cephalosporin, *ESBL* extended-spectrum beta-lactamase, *ESBL-PE* ESBL producing *Enterobacterales,* *ICU* intensive care unit

Out of 5993 patients admitted in ICU over the study period, 150 presented with a cefoxitin-susceptible ESBL-PE infection, and 41 patients were treated with cefoxitin during 24 h or more (Fig. 1). Additional file 1: Table S1 displays the 41 patients included in the study and the 105 patients treated with alternative treatments over the study period in the center and who were not included.

Baseline characteristics of the patients are described in Table 1. Twenty-four patients (59%) were under invasive mechanical ventilation and 22 (54%) were receiving vasopressors. The most common ESBL-PE infection cause was pneumonia (35 patients, 85%), and the most common pathogen was *Klebsiella pneumoniae* (25 patients, 61%). Cefoxitin MIC was estimated with Vitek2 in 30 patients. It was measured with *E*-test in 11 patients and amounted to 3 [2–4] mg/L. A probabilistic antibiotherapy was administered prior to inclusion in 29 patients (71%), with a median duration of 48 [24–72] h.

**Table 1:** Baseline characteristics

Variables	Median [IQR] or counts (%)
Age—years	59 [53–74]
Male sex	31 (76%)
Charlson comorbidity index	4 [3–8]
Immunocompromised patients	17 (41%)
Body weight—kg	65 [59–83]
SAPS 2 at ICU admission	48 [38–66]
SOFA score at ICU admission	7 [5–12]
Length of ICU stay before inclusion - days	10 [3–16]
Patients without renal replacement therapy	33 (80%)
Creatinine—µmol/L	82 [52–129]
CCR—mL/min	82 [43–90]
Patients with CCR ≥ 100 mL/min	15/33 (45%)
Patients under renal replacement therapy	8 (20%)
Continuous renal replacement therapy	8/8 (100%)
Ultrafiltration rate—mL/h	2000 [1925–2300]
Blood flow rate—mL/min	250 [250–250]
Length of ICU stay before inclusion—days	10 [3–16]
SOFA score at inclusion	8 [6–12]
Sepsis at inclusion	36 (88%)
Septic shock at inclusion	16 (39%)
Organ support techniques at inclusion	
Invasive mechanical ventilation	24 (59%)
Vasopressors	22 (54%)
Renal replacement therapy	8 (20%)
Extracorporeal membrane oxygenation	3 (7%)
Site(s) of infection with ESBL-PE^a^	
Respiratory tract	35 (85%)
Urinary tract	3 (7%)
Catheter related infection	3 (7%)
Ascites infection	1 (2%)
Bloodstream infection	11 (27%)
ESBL-PE species^b^	
* Klebsiella pneumoniae*	25 (61%)
* Escherichia coli*	14 (34%)
* Klebsiella oxytoca*	2 (5%)
* Citrobacter koseri*	1 (2%)
Cefoxitin susceptibility	
Assessed by Vitek2 only	30 (73%)
Assessed by *E*-test	11 (27%)
MIC for cefoxitin	
Vitek2 estimated MIC values (measured)—mg/L	
≤ 4 mg/L	36 (88%)
> 4 mg/L and ≤ 8 mg/L	5 (12%)
* E*-test measured and imputed MIC^c^ values—mg/L	3 [2–4]
Probabilistic antibiotherapy before definitive cefoxitin treatment	29 (71%)
Appropriate	24/29 (83%)
Inappropriate	5/29 (17%)
Duration of probabilistic antibiotherapy—h	48 [24–72]
Cefoxitin loading dose administered—g	41 (100%)
2 g	39 (95%)
4 g	2 (5%)
Maintenance administration of cefoxitin	
Continuous	39 (95%)
Intermittent	2 (5%)
Initial daily maintenance dose of cefoxitin—g	6 [6–6]
2 g	1 (2%)
3 g	1 (2%)
4 g	6 (15%)
6 g	26 (64%)
8 g	6 (15%)
12 g	1 (2%)
Duration of cefoxitin treatment—days	5 [4–7]

^a^Could be multiple

^b^Two species were identified in one patient

^c^Mixing *E*-test measured values when available, and imputed values for left-censored Vitek2-estimations in patients with missing E-test measured values

*CCR* creatinine clearance, *ESBL-PE* extended-spectrum beta-lactamase-producing *Enterobacterales*, *ICU* intensive care unit, *MIC* minimum inhibitory concentration, *SAPS 2* simplified acute physiology score 2, *SOFA* simplified organ failure assessment, *TDM* therapeutic drug monitoring

### Cefoxitin administration and PK

All patients received a median loading dose amounting to 2 [2] g, followed by a continuous administration with a median dose of 6 [6] g/24 h in 39 patients. Intermittent administration was performed in 2 patients. Seventy-two cefoxitin measured serum concentrations were available. On the first TDM occasion, sampling was performed at a median time of 19 [13–24] hours after cefoxitin bolus. The median total cefoxitin serum concentration was 37 [22–72] mg/L, 7 patients (18%) had a cefoxitin serum concentration over 100 mg/L and 9 patients (22%) did not reach the target serum concentration.

The final PK model was a one-compartment model including CCR_IBW_ and RRT as covariates influencing cefoxitin PK. The final model parameters are provided in Additional file 2: Table S2. Typical value of cefoxitin volume of distribution could not be reliably estimated. Although realistic (19.5 L), the estimate was associated with a high standard error (59%). It was therefore fixed at 12 L in the final model, in accordance with the results from Isla et al. [41]. Cefoxitin clearance in patients receiving RRT was estimated at 2.38 L/h. This clearance is similar to that of a patient with CCR_IBW_ of 27 mL/min. The model adequately described the data as shown in Additional file 3: Figure S1 and Additional file 4: Figure S2. Table 2 shows cefoxitin TDM and PK modeled values. The cefoxitin PK/PD target serum concentration of efficacy (*f*T_>MIC_ = 100%) was achieved in 34 patients (83%), based on model predictions (PK profile based on the Bayesian posterior estimates). However, only 19 (46%) patients achieved the PK/PD target *f*T_>MIC ECOFF_ = 100% (C_ss_ ≥ 5 × MIC_ECOFF_ in the case of continuous IV administration).

**Table 2:** Cefoxitin TDM and PK modeling

Variables	Median [IQR] or counts (%)
TDM—measured cefoxitin serum concentrations	
Delay between cefoxitin bolus and first TDM—h	19 [13–24]
Total serum cefoxitin concentration measured at 1st TDM—mg/L	37 [22–72]
Number of TDM occurrences per patient	
1	18 (44%)
2	15 (36%)
3	8 (20%)
Model-based cefoxitin serum concentrations 24 h after bolus	
Total serum cefoxitin concentration—mg/L	43 [23–72]
Patients with *f*T_>MIC_ = 100%	34 (83%)
Patients with *f*T_>MIC ECOFF_ = 100%	19 (36%)

*fT*_*>MIC*_ time spent above the MIC for the modeled free serum concentration, *IQR* interquartile range, *PK* pharmacokinetics, *MIC* minimal inhibitory concentration, *MIC*_*ECOFF*_ epidemiological cut-off value of minimal inhibitory concentration, *TDM* therapeutic drug monitoring

Results of the dosing simulations are shown in Additional file 5: Figure S3 and Additional file 6: Figure S4. Dosage requirements strongly depended on renal function. The standard dosage of 6 g/24 h administered by continuous IV was not sufficient to achieve the PK/PD targets for MIC up to 4–8 mg/L, except in patients with severe renal impairment or receiving continuous RRT. In patients with normal renal function, e.g., with CCR_IBW_ of 100 mL/min, only doses ≥ 10 g/24 h were associated with acceptable PTA for MIC up to 4 mg/L, while no tested regimen would have achieved acceptable PTA for a putative MIC = MIC_ECOFF_ (8 mg/L).

### Clinical outcomes

Clinical outcomes are provided in Table 3. Follow-up was completed until day-30 in all 41 patients. Median duration of cefoxitin treatment as definitive antibiotherapy was 5 [4–7] days.

**Table 3:** Outcomes

Variables	Median [IQR] or counts (%)
Cefoxitin treatment failure^a^	26 (63%)
Death	12 (29%)
Change of antibiotic before scheduled end of cefoxitin treatment	13 (32%)
Relapse of the infection with the same ESBL-PE	11 (27%)
Cefoxitin treatment failure	
Definitely linked to the initial ESBL-PE	14 (34%)
Possibly linked to the initial ESBL-PE	8 (19%)
Not linked to the initial ESBL-PE	4 (10%)
Delay between inclusion and death—days	8 [5–14]
Delay between inclusion and relapse with the same ESBL-PE—days	10 [5–20]
Acquisition of cefoxitin-resistance with the same species of ESBL-PE	13 (32%)
Infection	4 (10%)
Carriage	9 (22%)
Delay between inclusion and identification of cefoxitin-resistance—days	11 [10–20]
Number of patients presenting a serious adverse event	7 (17%)
Nature of adverse event^a^	
Acute kidney injury	4 (10%)
Rash	2 (5%)
Hepatic cytolysis	1 (2%)
Encephalopathy	1 (2%)
Cholestasis	0
Cefoxitin discontinuation due to serious adverse event	1 (2%)
Occurrence of *Clostridioides difficile* infection	2 (5%)

^a^Could be multiple

*ESBL-PE* extended-spectrum beta-lactamase-producing *Enterobacterales*, *IQR* interquartile range

#### Cefoxitin treatment failures

During follow-up, treatment failure occurred in 26 patients (63%). Neither the ESBL-PE species, nor the cefoxitin MIC were associated with treatment failure (Additional file 7: Table S3). Criteria of failure could be multiple per patient and were as follows: death (12 patients, 29%) with a median of 8 [5–14] days after inclusion, change of antibiotic before the scheduled end of cefoxitin treatment course (13 patients, 32%), and relapse of infection with the same ESBL-PE with a median of 10 [5–20] days after inclusion (11 patients, 27%). Death was possibly linked to the initial ESBL-PE in 3 cases, and not linked in 9 cases (among them, 7 non-infectious causes). The change of cefoxitin for another antibiotic was definitely linked to the first pathogen in 4 cases, possibly linked in 4 cases, and not linked in 5 cases (among them, 1 case of cefoxitin-associated adverse event).

#### Cefoxitin safety

Serious adverse events attributed to a drug side effect occurred in seven patients (17%). Cefoxitin-associated adverse events were responsible for anticipated end of treatment in one patient (2%). No death was attributed to a drug side effect. Sixteen patients (39%) presented one or more other infections during the follow-up period, among whom 2 patients (5%) presented with *Clostridioides difficile* infection.

#### Impact of cefoxitin exposure on cephamycin-susceptibility

Acquisition of cefoxitin-resistance with the same bacteria during follow-up was found in 13 cases (32%), including 4 infections and 9 asymptomatic carriages. The rate of acquisition of cefoxitin resistance was 7/14 (50%) for *Escherichia coli,* 5/25 (20%) for *Klebsiella pneumoniae*, and 1/2 (50%) for *Klebsiella oxytoca.* Low cefoxitin serum concentration at first TDM occasion was associated with the emergence of cefoxitin-resistance (*p* = 0.008, Additional file 8: Table S4).

## Discussion

To our knowledge, this is the first study having evaluated the PK/PD of cefoxitin, its clinical efficacy and tolerance in critically ill patients. The main findings of the study were: (1) cefoxitin displayed a large interindividual PK variability in ICU patients as expected, and continuous administration of high doses is required to achieve the PK/PD target; (2) the PTA is unacceptably low for MIC ≥ 4 mg/L and normal or increased renal function, suggesting that the cefoxitin susceptibility breakpoint for *Enterobacterales* is inappropriate; (3) the failure rate of cefoxitin treatment in severe ESBL-PE infections in ICU was 63%; (4) a high rate of cefoxitin resistance emergence under treatment was observed (32%) and was significantly associated with cefoxitin underdosage.

### Optimal mode of administration and PK in ICU

Cefoxitin is compatible with a wide variety of commonly used infusion solutions and is stable for continuous administration for 40 h at 25 °C [45]. Continuous administration appears to be especially relevant for cefoxitin, considering its time-dependent activity and its very short half-life, i.e., 40 min in patients with normal renal function [8, 37, 46]. In our study, cefoxitin was administered by continuous infusion with a syringe pump after an initial bolus in most patients, and we observed PK features consistent with previous studies. Cefoxitin clearance was influenced by renal function and RRT. We observed a quasi-linear relationship between cefoxitin clearance and CCR, and cefoxitin clearance was higher than CCR, as reported previously [41, 46, 47], consistent with cefoxitin renal elimination via both glomerular filtration and active tubular secretion.

**Fig. 2:**
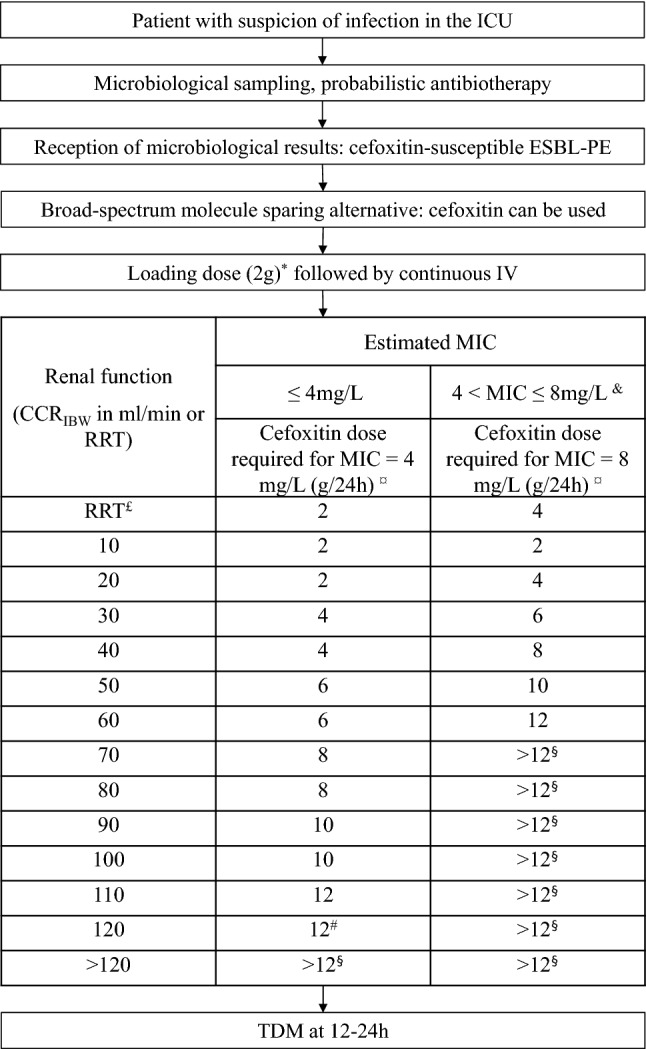
Proposed decision and posologic algorithm for cefoxitin use in ICU patients with ESBL-PE infection. ^*^Proposed loading dose and consider measuring the MIC with *E*-test in this situation. ^¤^Cefoxitin dosages required for achieving PTA ≥ 90% for target 100% *f*T_>MIC_ at 48 h, considering the maximal possible value for MIC in this interval of estimation (i.e., 4 mg/L (left) and 8 mg/L (right)). ^£^Considering a continuous RRT and using standard parameters (blood flow rate 250 mL/min, ultrafiltration rate 2000 mL/h). ^#^The computed PTA for CCR_IBW_ = 120 mL/min and a dose of 12 g/24 h was 89.5%, which was rounded to 90% and considered as acceptable. ^§^The use of a dosage of cefoxitin above 12 g/24 h has not been reported so far. The absence of data concerning toxicity and uncertainty considering the possible non-linear PK over such dosage should be kept in mind. *CCR*_*IBW*_ creatinine clearance based on ideal body weight, *ICU* intensive care unit, *ESBL-PE* extended-spectrum beta-lactamase producing *Enterobacterales*, *fT*_>MIC_ proportion of time with free cefoxitin serum concentration over the minimum inhibitory concentration, *MIC* minimum inhibitory concentration, *PK *pharmacokinetics, *PTA* probability of target attainment, *TDM* therapeutic drug monitoring, *RRT* renal replacement therapy, *ICU* intensive care unit

Our PK results identified a very large variability of cefoxitin PK in critically ill patients, as expected. PK/PD simulations showed that dosage requirements strongly depend on renal function and bacterial MIC. The standard doses of cefoxitin (6 g/24 h) appeared insufficient to achieve PK targets, which is consistent with prior findings in the medical settings [48]. Indeed, doses up to 12 g/24 h appear necessary to achieve the recommended beta-lactam PK/PD target (*f*T_>MIC_ = 100%) in critically ill patients for MIC up to 4 mg/L in patients with normal renal function. Even larger doses would be necessary in patients with augmented renal function and/or higher MIC, which raises safety concerns, as doses larger than 12 g/24 h have not been clinically evaluated, to our knowledge. Our results are consistent with those from Isla et al. [41] in the perioperative setting, who reported that cefoxitin should be administered every hour to maintain serum free concentration above 8 mg/L in patients with CCR of 100 mL/min. Continuous IV administration, high doses, dose adjustments on renal function, and MIC determination appear necessary to optimize cefoxitin PK/PD in ICU patients, and therefore consider cefoxitin as a treatment for ESBL-PE in ICU patients, as suggested earlier in other settings [46] or with other beta-lactams [36]. However, cefoxitin dosage based on PK/PD simulations are set to maximize PTA and may not be suited for all patients, as they may be associated with too high serum concentrations in some individuals. TDM is required to avoid overexposure when initial cefoxitin doses are chosen based on PK/PD simulations. We propose here an algorithm for guidance in the use of cefoxitin in ICU settings, based on our PK modeling (Fig. 2).

### Cefoxitin efficacy in ICU

Previous studies have reported a high success rate of cefoxitin treatment in small series of non-ICU patients with urinary tract infections [14–17]. Apart from this indication, studies assessing cefoxitin efficacy are scarce but reported a high rate of success, ranging from 85 to 95% depending on the site of infection [18]. A previous study comparing cefmetazole—another cephamycin—and carbapenems showed a high rate of success in the treatment of ESBL-PE bacteremia [19]. However, higher failure rates are expected in the ICU setting.

As alternatives for the treatment of ESBL-PE, cephamycins have rarely been compared with carbapenems [20, 42, 43] and exclusively in non-ICU patients. In the present study, cefoxitin failure rate against ESBL-PE infections was 63%, slightly higher than the carbapenems or piperacillin–tazobactam failure rates reported for severe ESBL-PE infections (54%) in a retrospective study on 107 critically ill patients, sharing a comparable definition of treatment failure [44]. Several reasons might explain this failure rate in the ICU setting. First, some patients might present a persistence of cultural growth of ESBL-PE after treatment that might be considered erroneously as a persistent infection. Secondly, some situations of clinical worsening under appropriate antibiotic therapy are sometimes wrongly attributed to an uncontrolled initial infection. Finally, mortality in this setting is often multifactorial (i.e., not always attributable to an uncontrolled infection) while included in the definition of failure in the above-mentioned studies, as it was the case in our study in 7 patients.

### Safety and tolerance

Cefoxitin use is deemed safe in many infectious situations [13]. In a small series of 38 miscellaneous infections, cefoxitin was well tolerated, with the exception of 4 cases of acute kidney injury, 6 cases of elevated eosinophilic cells counts, and burning feeling at intravenous site during infusion [18]. Case reports reported the following complications: hemolytic anemia [49], exfoliative dermatitis, neutropenia and *Clostridioides difficile* colitis [50]. Former data identified good renal tolerance of cefoxitin, even in patients with chronic kidney disease [51]. However, most data were observed in the perioperative prophylactic setting [21]. To our knowledge, no toxicity serum concentration threshold has been defined for cefoxitin so far. Limited information exists on the tolerance of high-dose cefoxitin. In patients treated for *Mycobacterium abscessus* infection with doses up to 12 g/24 h, tolerance was described as moderate to poor, with a high rate of hematologic toxicity (51%) and hepatotoxicity (15%) [52]. However, these patients also received other antimicrobial agents, questioning the direct involvement of cefoxitin in observed toxicity. In our study, acute kidney injury was relatively frequent, but critically ill patients often combine multiple risk factors for acute kidney injury (not only drug exposure). *Clostridioides difficile* infection occurrence was low, in contrast with previous reports [50]. Overall, clinical tolerance of cefoxitin in the ICU appeared good, even in patients with cefoxitin serum concentration over 100 mg/L.

### Impact of cefoxitin exposure on cephamycin-susceptibility

The ecological impact of cefoxitin is supposed to be lower than that of carbapenems. However, it may not be negligible, since acquisition of cefoxitin-resistance has been reported, specifically in *Klebsiella pneumonia* and related to porin deficiency [23]. As a consequence, a lower efficacy is expected in the treatment of *Klebsiella pneumonia* infections compared with other *Enterobacterales* such as *Escherichia coli* [22, 23, 25]. This caution is debated though, as such a mechanism has also been found in Escherichia coli [24]. Furthermore, some authors found cefoxitin equally effective in both *Escherichia coli* and *Klebsiella pneumoniae* urinary tract infections with a low rate of resistance acquisition [15]. In our study, acquisition of resistance was quite frequent, and was associated with low exposure. However, *Klebsiella pneumonia* was not significantly associated with acquisition of cefoxitin-resistance (Additional file 8: Table S4)*,* questioning the relevance of this theoretical risk of lower efficacy of cefoxitin in *Klebsiella pneumoniae* infections related to porin loss. Nevertheless, the low statistical power of our study may explain this finding. Furthermore, as the antibiogram for ESBL-PE found in fecal swab was not systematically performed during follow-up (it was systematically performed for ESBL-PE found in any other biological samples though), the rate of resistance acquisition might have been under-evaluated in our study.

### Limitations and strengths

Our study has several limitations. Its retrospective design may have induced an information bias. The lack of a control group precludes firm conclusions regarding clinical efficacy and safety. Nevertheless, a large previous study regarding the efficacy of carbapenems in ICU ESBL-PE infections [44] was consistent with our findings in terms of failure rate. As only 30% patients presenting with cefoxitin-susceptible ESBL-PE infection in our center were treated with cefoxitin, a selection bias cannot be ruled out. Regarding TDM and PK analysis, the sampling was sparse and not performed at the same time for all patients.

Nevertheless, our study has several strengths: this is one of the largest cohorts of patients treated with cefoxitin in the ICU reported so far. Furthermore, this study is the first providing PK data on cefoxitin in the ICU setting. In addition, we provide the first dose recommendations in critically ill patients based on PK modeling, which may be helpful for clinicians. Also, the PK analysis provides data that might be used for questioning the relevance of the susceptibility breakpoint for cefoxitin. Finally, this study may help designing a complementary prospective comparative study so as to determine whether cefoxitin is an acceptable alternative for carbapenems.

## Conclusion

In this study performed in critically ill patients, continuous administration of large doses of cefoxitin appeared necessary to achieve the recommended beta-lactam PK/PD target in patients with normal renal function, at the price of unnecessary high serum concentrations in some patients. Renal status, MIC determination and TDM may be useful for dosage individualization in this setting. The cefoxitin safety profile was favorable, but we observed a high rate of cephamycin-resistance emergence.

## Supplementary Information

**Additional file 1: Table S1.** Characteristics and outcome of patients presenting with cefoxitin-susceptible-ESBL-PE infections admitted in the ICU during the study period.

**Additional file 2:** **Table S2.** Population PK parameters of cefoxitin.

**Additional file 3:** **Figure S1.** Model prediction versus observed cefoxitin concentrations.

**Additional file 4:** **Figure S2.** Visual predictive check obtained with the final model.

**Additional file 5: Figure S3. **Probability of target attainment for various cefoxitin doses in patients with CCRIBW = 100 ml/min.

**Additional file 6: Figure S4. **Probability of target attainment for different levels of renal function with a cefoxitin dose of 6g/day.

**Additional file 7: Table S3. **Univariate factors associated with cefoxitin treatment failure.

**Additional file 8: Table S4. **Univariate factors associated with the acquisition of cefoxitin-resistance.

## Data Availability

The datasets generated and analyzed during the current study are not publicly available, but are available from the corresponding author on reasonable request.

## Abbreviations

ABW: Adjusted body weight
CCR: Creatinine clearance
C_SS_: Steady-state total serum concentration
ESBL-PE: Extended-spectrum beta-lactamase-producing *Enterobacterales*
MIC_ECOFF_: Epidemiological cut-off minimum inhibitory concentration
*f*C_SS_: Steady-state free serum concentration
*f*T_>MIC_: Proportion of time spent above the minimum inhibitory concentration
IBW: Ideal body weight
ICU: Intensive care unit
MIC: Minimum inhibitory concentration
PK: Pharmacokinetics
PK/PD: Pharmacokinetics/pharmacodynamics
PTA: Probability of target attainment
RRT: Renal replacement therapy
SAPS: Simplified Acute Physiology Score
SOFA: Sequential Organ Failure Assessment
TBW: Total body weight
TDM: Therapeutic drug monitoring
