# Supplementary material for: Pharmacokinetics, efficacy and tolerance of cefoxitin in the treatment of cefoxitin-susceptible extended-spectrum beta-lactamase producing Enterobacterales infections in critically ill patients: a retrospective single-center study
Source: Ann Intensive Care. 2022 Sep 30;12:90. doi: 10.1186/s13613-022-01059-9 (PMC9522958; doi:10.1186/s13613-022-01059-9)
Supplement: Supplementary file 1 — Additional file 1: Table S1. Characteristics and outcome of patients presenting with cefoxitin-susceptible-ESBL-PE infections admitted in the ICU during the study period. [file 13613_2022_1059_MOESM1_ESM.docx]

Additional - Table 1. Characteristics and outcome of patients presenting with cefoxitin-susceptible-ESBL-PE infections admitted in the ICU during the study period.

| Variables | Adapted antibiotherapy | |
| --- | --- | --- |
|  | Cefoxitin (41 patients, included) | Alternative antibiotic treatments (105 patients, non-included) |
| Age − years | 59 [53-74] | 67 [56-74] |
| Male sex | 31 (76%) | 65 (62%) |
| SAPS 2 at ICU admission | 48 [38-66] | 42 [34-52] |
| Probabilistic antibiotherapy administered and effective against the initial ESBL-PE | 24/41 (59%) | 71/98^¤^ (72%) |
| Length of ICU stay before initiation of adapted antibiotherapy^#^ | 10 [3-16] | 8 [3 – 17] |
| Site(s) of infection^*^ |  |  |
| - Respiratory tract | 35 (85%) | 66 (63%) |
| - Urinary tract | 3 (7%) | 27 (26%) |
| - Bloodstream infection | 3 (7%) | 23 (21%) |
| - Other | 11 (27%) | 5 (5%) |
| ESBL-PE species^*^ |  |  |
| - *Klebsiella pneumoniae* | 25 (61%) | 75 (71%) |
| - *Escherichia coli* | 14 (34%) | 41 (39%) |
| - *Klebsiella oxytoca* | 2 (5%) | 6 (6%) |
| - *Citrobacter koseri* | 1 (2%) | 0 (0%) |
| Day 30 mortality from time of initiation of adapted antibiotherapy^#^ | 12/41 (29%) | 33/98^¤^ (34%) |

Values are count (percentage) or median [IQR]

^*^ could be multiple

^¤^ 7 subjects lost to follow-up

^#^ the time of initiation of adapted antibiotherapy was the day of inclusion (group cefoxitin) or the day of issuance of the antibiotic susceptibility testing of the first biological sample positive for ESBL-PE defining the infection (*i.e.* 48h after sampling)

ESBL-PE, extended spectrum betalactamase-producing *Enterobacterale*; ICU, intensive care unit; IQR, interquartile range; SAPS 2, simplified acute physiology score 2
