# Supplementary material for: Pharmacokinetics, efficacy and tolerance of cefoxitin in the treatment of cefoxitin-susceptible extended-spectrum beta-lactamase producing Enterobacterales infections in critically ill patients: a retrospective single-center study
Source: Ann Intensive Care. 2022 Sep 30;12:90. doi: 10.1186/s13613-022-01059-9 (PMC9522958; doi:10.1186/s13613-022-01059-9)
Supplement: Supplementary file 2 — Additional file 2: Table S2. Population PK parameters of cefoxitin. [file 13613_2022_1059_MOESM2_ESM.docx]

Additional - Table 2. Population PK parameters of cefoxitin

| Variables | Value | Relative standard error (%) |
| --- | --- | --- |
| *Fixed effects* | | |
| V_pop_ (L) | 12 (fixed) | NA |
| CL_pop_ (L/h)^1^ | 10 | 11.7 |
| β_CCR_IBW_^1^ | 1.09 | 0.153 |
| CL_RRT_ (L/h)^1^ | 2.38 | 8.82 |
| *Standard deviation of the random effects* | | |
| omega_V | 1.12 | 32.4 |
| omega_CL_pop_ | 0.558 | 15.5 |
| *Error model parameters (proportional error)* | | |
| β | 0.361 | 11.4 |

1: Typical clearance is as follows: CL=CL_pop_.(CCR_IBW_/100)^βCCR_IBW^ in patients without RRT ; CL=CL_RRT_ in patients with RRT.

CL, total cefoxitin clearance; CL_pop,_ population typical value for clearance; CCR_IBW_, creatinine clearance based on ideal body weight; NA, not applicable; PK, pharmacokinetics; RRT, renal replacement therapy; V, distribution volume; V_pop_, population typical value for distribution volume.
