# Supplementary material for: Pharmacokinetics, efficacy and tolerance of cefoxitin in the treatment of cefoxitin-susceptible extended-spectrum beta-lactamase producing Enterobacterales infections in critically ill patients: a retrospective single-center study
Source: Ann Intensive Care. 2022 Sep 30;12:90. doi: 10.1186/s13613-022-01059-9 (PMC9522958; doi:10.1186/s13613-022-01059-9)
Supplement: Supplementary file 3 — Additional file 3: Figure S1. Model prediction versus observed cefoxitin concentrations. [file 13613_2022_1059_MOESM3_ESM.pdf]

Additional Figure 1. Model prediction *versus* observed cefoxitin concentrations

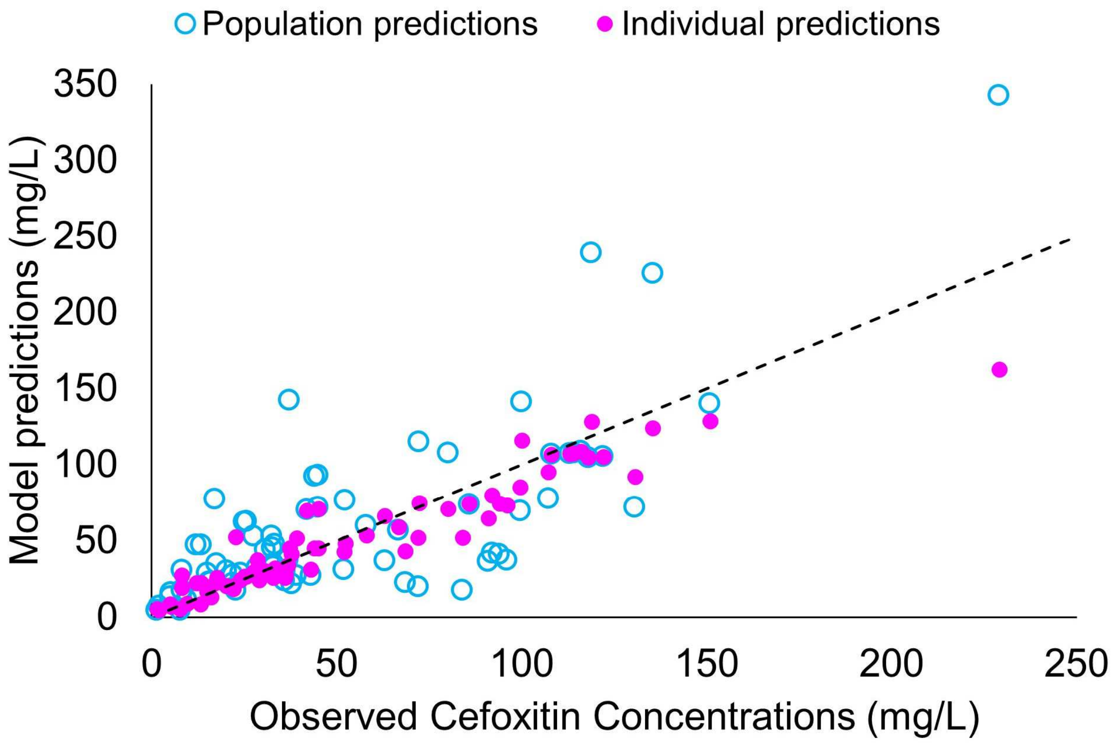

R^2 = 0.91; Mean prediction error (bias): -3.5 mg/l; Median absolute error (imprecision): 14.5 %

The dashed line is the line of identity (y = x)
