# Supplementary material for: Pharmacokinetics, efficacy and tolerance of cefoxitin in the treatment of cefoxitin-susceptible extended-spectrum beta-lactamase producing Enterobacterales infections in critically ill patients: a retrospective single-center study
Source: Ann Intensive Care. 2022 Sep 30;12:90. doi: 10.1186/s13613-022-01059-9 (PMC9522958; doi:10.1186/s13613-022-01059-9)
Supplement: Supplementary file 4 — Additional file 4: Figure S2. Visual predictive check obtained with the final model. [file 13613_2022_1059_MOESM4_ESM.pdf]

Additional Figure 2. Visual predictive check obtained with the final model

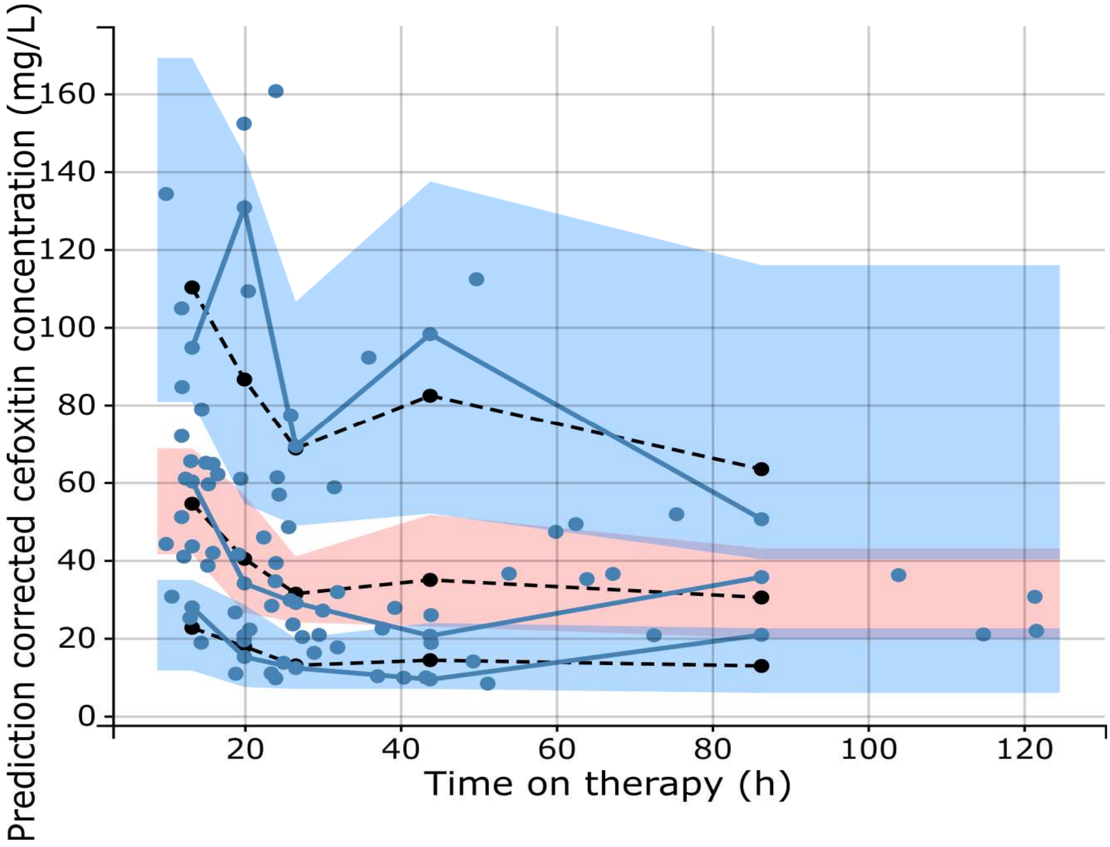

Blue dots are prediction-corrected cefoxitin concentrations. Blue solid lines are from bottom to top the simulated 10th, 50th (median), and 90th percentiles, respectively. Black dotted lines are the corresponding empirical percentiles. The blue areas display the 90% confidence interval of the simulated 10th and 90th percentiles, while the pink area depicts the 90% confidence interval of the simulated median.
