# Supplementary material for: Pharmacokinetics, efficacy and tolerance of cefoxitin in the treatment of cefoxitin-susceptible extended-spectrum beta-lactamase producing Enterobacterales infections in critically ill patients: a retrospective single-center study
Source: Ann Intensive Care. 2022 Sep 30;12:90. doi: 10.1186/s13613-022-01059-9 (PMC9522958; doi:10.1186/s13613-022-01059-9)
Supplement: Supplementary file 5 — Additional file 5: Figure S3. Probability of target attainment for various cefoxitin doses in patients with CCRIBW = 100 ml/min. [file 13613_2022_1059_MOESM5_ESM.pdf]

Additional Figure 3. Probability of target attainment for various cefoxitin doses in patients with CCR_{IBW} = 100 ml/min

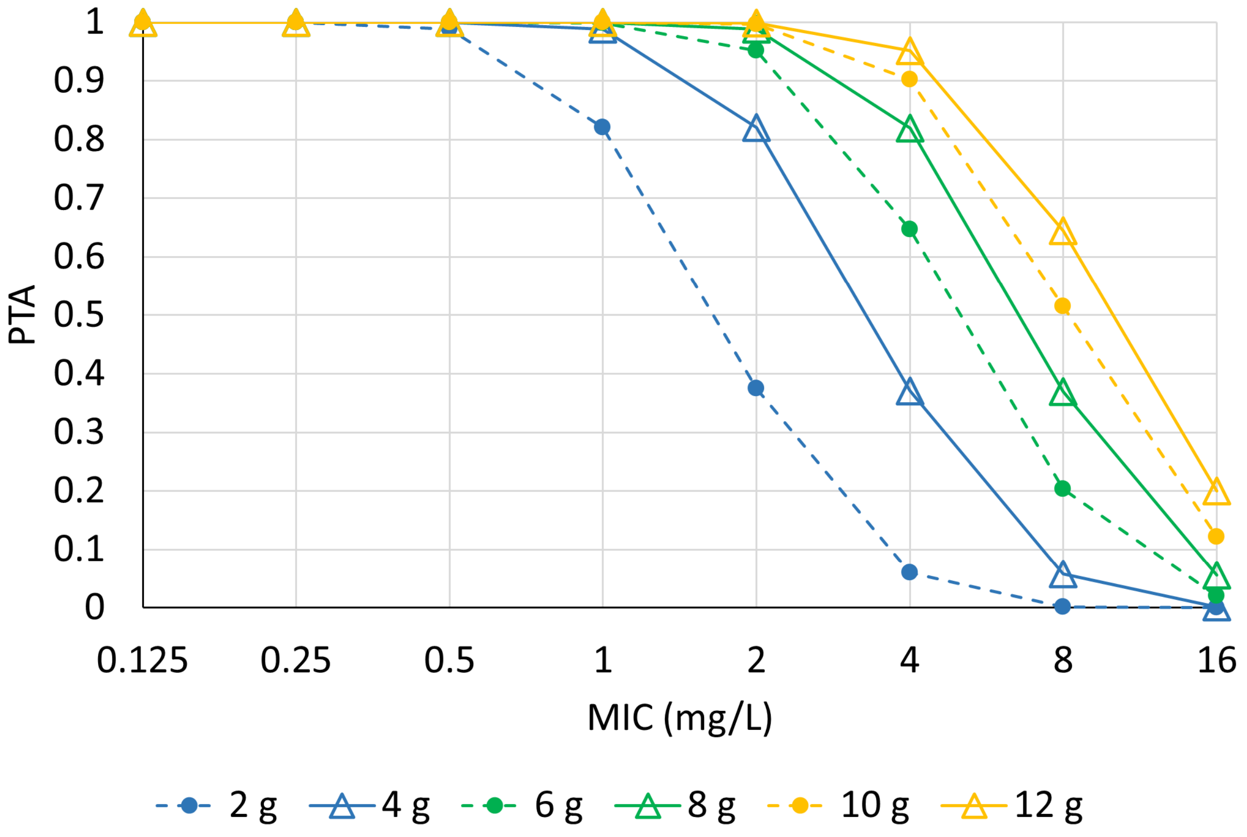

Simulated continuous IV administration of cefoxitin with daily doses ranging from 2 to 12 g, after a loading dose of 2 g administered over 1h.

CCR_{IBW}, creatinine clearance based on ideal body weight; MIC, minimum inhibitory concentration; PTA, probability of target attainment
