# Supplementary material for: Pharmacokinetics, efficacy and tolerance of cefoxitin in the treatment of cefoxitin-susceptible extended-spectrum beta-lactamase producing Enterobacterales infections in critically ill patients: a retrospective single-center study
Source: Ann Intensive Care. 2022 Sep 30;12:90. doi: 10.1186/s13613-022-01059-9 (PMC9522958; doi:10.1186/s13613-022-01059-9)
Supplement: Supplementary file 6 — Additional file 6: Figure S4. Probability of target attainment for different levels of renal function with a cefoxitin dose of 6g/day. [file 13613_2022_1059_MOESM6_ESM.pdf]

Additional Figure 4. Probability of target attainment for different levels of renal function with a cefoxitin dose of 6g/day

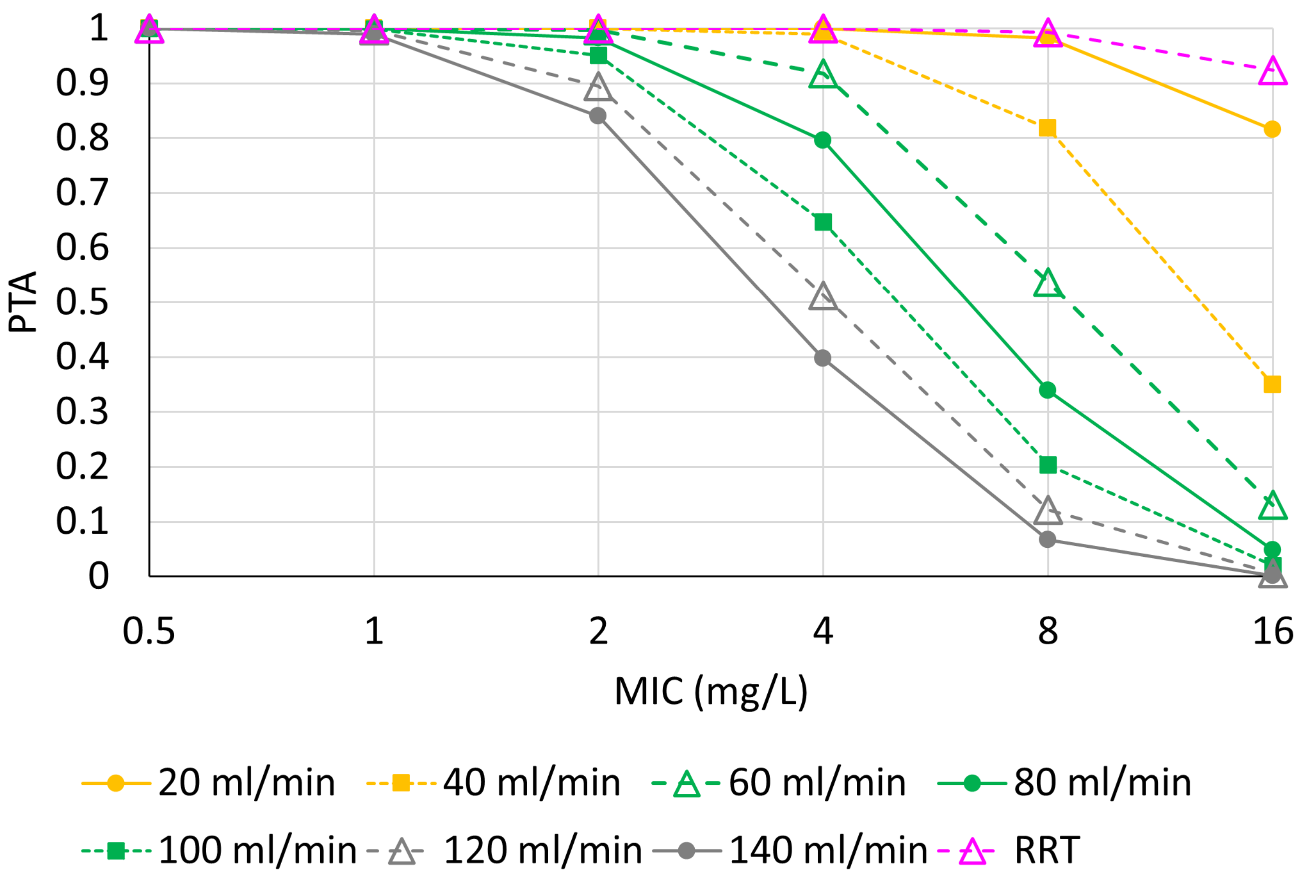

Simulated continuous IV administration of cefoxitin with a daily dose of 6 g, after a loading dose of 2 g administered over 1h.

Renal function is based on CCR_{IBW}. RRT is considered continuous RRT using standard parameters (blood flow rate 250ml/min, ultrafiltration rate 2000ml/h)

CCR_{IBW}, creatinine clearance based on ideal body weight; MIC, minimum inhibitory concentration; PTA, probability of target attainment; RRT, renal replacement therapy
