# Supplementary material for: Pharmacokinetics, efficacy and tolerance of cefoxitin in the treatment of cefoxitin-susceptible extended-spectrum beta-lactamase producing Enterobacterales infections in critically ill patients: a retrospective single-center study
Source: Ann Intensive Care. 2022 Sep 30;12:90. doi: 10.1186/s13613-022-01059-9 (PMC9522958; doi:10.1186/s13613-022-01059-9)
Supplement: Supplementary file 7 — Additional file 7: Table S3. Univariate factors associated with cefoxitin treatment failure. [file 13613_2022_1059_MOESM7_ESM.docx]

Additional - Table 3. Univariate factors associated with cefoxitin treatment failure

| Explanatory variables | Outcome | | *p* value |
| --- | --- | --- | --- |
|  | Treatment success  (n=15) | Treatment failure (n=26) |  |
| Age - years | 59 [55-69] | 59 [52-79] | 0.511 |
| Male sex | 12 (80%) | 19 (73%) | 0.720 |
| Immunocompromised patient | 6 (40%) | 11 (42%) | 1.000 |
| Adapted probabilistic antibiotherapy | 12 (80%) | 11 (42%) | 0.183 |
| *Klebsiella pneumoniae*  Cefoxitin MIC [4 - 8 mg/L] | 9 (60%)  3 (20%) | 16 (62%)  2 (8%) | 1.000  0.336 |
| Sepsis at the time of inclusion | 13 (87%) | 20 (77%) | 0.691 |
| SOFA score at the time of inclusion | 7 [6-11] | 8 [6-13] | 0.471 |
| 24 hours after bolus (modeling values)   - Total serum cefoxitin concentration - mg/L - Patients with cefoxitin concentration > 5*MIC | 35 [23-80]  11 (73%) | 43 [23-69]  23 (88%) | 0.978  0.207 |

Values are count (percentage) or median [IQR].

ICU, Intensive Care Unit; IQR, interquartile range; MIC, Minimum Inhibitory Concentration; SOFA, Sequential Organ Failure Assessment.
