# Supplementary material for: Pharmacokinetics, efficacy and tolerance of cefoxitin in the treatment of cefoxitin-susceptible extended-spectrum beta-lactamase producing Enterobacterales infections in critically ill patients: a retrospective single-center study
Source: Ann Intensive Care. 2022 Sep 30;12:90. doi: 10.1186/s13613-022-01059-9 (PMC9522958; doi:10.1186/s13613-022-01059-9)
Supplement: Supplementary file 8 — Additional file 8: Table S4. Univariate factors associated with the acquisition of cefoxitin-resistance. [file 13613_2022_1059_MOESM8_ESM.docx]

Additional - Table 4. Univariate factors associated with the acquisition of cefoxitin-resistance

| Explanatory variable | Acquisition of cefoxitin-resistance  (n=13) | No cefoxitin-resistance  (n=28) | *p*-value |
| --- | --- | --- | --- |
| Previous antibiotic treatment | 11 (85%) | 19 (68%) | 0.451 |
| Respiratory tract infection | 13 (100%) | 22 (79%) | 0.152 |
| Duration of cefoxitin treatment - days | 5 [4-6] | 5 [4-7] | 0.590 |
| **Cefoxitin concentration (measured) at first TDM – mg/L** | 22 [10-29] | 49 [33-102] | **0.008** |
| *Klebsiella pneumoniae*  Cefoxitin initial MIC – mg/L | 5 (38%)  2 [2-3] | 20 (71%)  3 [3-4] | 0.084  0.074 |

Values are count (percentage) or median [IQR]

IQR, interquartile range; MIC, minimum inhibitory concentration; TDM, therapeutic drug monitoring
